# Death of Dr. Few, of South Carolina

**Published:** 1885-04-20

**Authors:** 


### DEATH OF DR. FEW.

We are pained to learn that Dr. T. S. Few, of South Carolina, the young gentleman who recently received the first honor at the late commencement of the Southern Medical College, was seized with Pneumonia soon after reaching his home in South Carolina, and died after a short illness. Dr. Few was a young man of many excellent qualities. By extraordinary study and diligence he so far overcame the disadvantages of a limited education as to secure the prize for the first honor in a large class of intelligent young gentlemen, and with high hope and brilliant anticipations for the future he left for his home to enter upon the duties of the Profession; but, like a flower nipped by the untimely frost, he was cut down and is now numbered with the departed. This mysterious Providence will bring sadness and sorrow to numerous college-mates and to many here, whose friendship he had secured by his kind and genial deportment.

